# Detecting Diseases in Medical Prescriptions Using Data Mining Tools and Combining Techniques

**Published:** 2016

**Authors:** Mehdi Teimouri, Farshad Farzadfar, Mahsa Soudi Alamdari, Amir Hashemi-Meshkini, Parisa Adibi Alamdari, Ehsan Rezaei-Darzi, Mehdi Varmaghani, Aysan Zeynalabedini

**Affiliations:** a *Department of Network Science and Technology, Faculty of New Sciences and Technologies, University of Tehran, Tehran, Iran. *; b *Non-communicable disease Research Center, Endocrinology and Metabolism Population Science Institute, Tehran University of Medical Sciences, Tehran, Iran. *; c *Department of Pharmacoeconomics, Faculty of Pharmacy, Tehran University of Medical Sciences, Tehran, Iran.*; d *School of medicine, Shahid Beheshti University of Medical Sciences, Tehran, Iran. *; e *Department of Epidemiology and Biostatistics, School of Public Health, Tehran University of Medical Sciences, Tehran, Iran.*; f *School of Medicine, Orumia University of Medical Sciences, Orumia, Iran.*

**Keywords:** Outpatient Diseases, Medical Prescription, Diagnosis, Data Mining; Voting, Weighted Voting, Stacking

## Abstract

Data about the prevalence of communicable and non-communicable diseases, as one of the most important categories of epidemiological data, is used for interpreting health status of communities. This study aims to calculate the prevalence of outpatient diseases through the characterization of outpatient prescriptions. The data used in this study is collected from 1412 prescriptions for various types of diseases from which we have focused on the identification of ten diseases. In this study, data mining tools are used to identify diseases for which prescriptions are written. In order to evaluate the performances of these methods, we compare the results with Naïve method. Then, combining methods are used to improve the results. Results showed that Support Vector Machine, with an accuracy of 95.32%, shows better performance than the other methods. The result of Naive method, with an accuracy of 67.71%, is 20% worse than Nearest Neighbor method which has the lowest level of accuracy among the other classification algorithms. The results indicate that the implementation of data mining algorithms resulted in a good performance in characterization of outpatient diseases. These results can help to choose appropriate methods for the classification of prescriptions in larger scales.

## Introduction

In order to design and implement national and international health policies, it is necessary to collect epidemiological data and use this information to interpret the health status of communities. One of the most important categories of epidemiological data is the data on the prevalence of communicable diseases and non-communicable diseases which is used to calculate the burden of diseases (1). In the previous studies conducted on the burden of diseases, hospital and mortality data is usually used to calculate the prevalence of diseases. But, in order to determine the burden of some diseases such as influenza, data from outpatient care centers along with hospital and mortality data should be used (3). Karl Berg and Elo have estimated burden of ischemic heart disease and coronary artery disease risk factors for a target population using data collected from all health services (including inpatient and outpatient care centers) (4). The results of this research indicate that to achieve a more comprehensive estimate of burden of diseases, not only the hospitalization data but also the data about outpatient care should be used. A similar study was carried out in 2012 by a group of Spanish researchers who pointed out the importance of outpatient data (5). Their study examines the prevalence of 12 chronic diseases. A range of sources, including electronic patient records, prescriptions, and hospitalization data are used for the diagnosis of diseases.

In another study, conducted in 2012 to determine the impact of Rota virus vaccination on the burden of diarrhea disease in Hindi and Alaska under-five-year children who are living in America, the researchers used outpatient data along with hospitalization data (6). In this study, data collected from 2001 to 2006 is used to determine the prevalence of diseases associated with diarrhea in children before the vaccination and data collected in 2008, 2009 and 2010 is used to determine the prevalence of the disease after the vaccination. All of these studies are conducted to assess burden of diseases. However, there is no mention of using classification methods in any of these studies. 

The prevalence of diseases can be calculated through categorizing prescriptions based on the type of disease. Since Iranian physicians do not fill up the field of “type of disease” in prescriptions, we should seek a solution to detect the disease in each prescription automatically. For this purpose, we took advantage of data mining tools. In recent years, many studies have been conducted on diagnosis of diseases and prescription making using data mining tools. For example, a study, which was conducted in 2011 in Hong Kong, creates a system for prescriptions making that combines association rules and Case-Based Reasoning (CBR). This system uses electronic patient records, lab results, and symptoms of patients for prescription making (7). In a similar study, Support Vector Machine and Neural Network methods are used to detect heart disease and prescribe medications for those conditions. To diagnose and prescribe appropriate medications, the system uses the data about patient records, laboratory test results, cardiovascular system status, nervous system function, blood pressure, respiratory system, and most importantly, electrocardiogram (ECG) (8). Data mining methods are not only applied to the mentioned methods. For example, in another study Decision Tree algorithm is used to determine the stage of Type II diabetes, treatment planning, and pharmaceutical dosage (9).

One of the challenges associated with drug administration is the presence or absence of interactions between prescribed drugs in a prescription. Drug interactions annually lead to millions of injuries, hospital stays, and deaths in the world and their related costs exceed hundreds of millions of dollars. Many studies have used data mining tools to avoid drug interactions. For example, in a study, Decision Tree is used to develop a decision support system for determining hospital stays related to adverse drug events and drug interactions (10). To create this system, 500 rules are extracted from 10500 cases; a number of extracted rules are approved by experts and the other rules are under the review. In another study Decision Tree is used to detect the effects of drug interactions on central nervous system, liver, and it also evaluated the allergic effects of 507 drugs; the study was carried out for determining the physical, chemical, and structural medicinal compounds which could lead to drug interactions (11). Also, a study was conducted in 2011 that uses Support Vector Machine and Logistic Regression to detect drug interactions (12). There are numerous studies in this field, which put emphasis on the importance of dealing with drug interactions (13), (14) and (15).

In this project we aim to help developing a system for detecting the disease in each prescription with minimum error using data mining tools, so that it would not require specialized manpower, considerable time, and money.

## Experimental

*Data*

From 2004 to date, 115 million prescriptions have been registered at the Iranian Ministry of Health, Food and Drug Administration that Non-communicable Disease Research Center of Tehran University attempts to get a copy of this data for researching purposes. In October 2013 the Ministry gave the non-communicable Disease Research Center a sample of 1412 prescriptions to check the data. These prescriptions are related to Babol University of Medical Sciences clinics that have been administered in June 2010 and all of prescriptions are insured by Social Security Organization and Health Insurance Organization. In this paper we used this sample to develop a model for diagnosing the diseases of prescriptions.

There are no data on the age and the gender and all of prescriptions are prescribed by general physicians. For this reason, only the names of drugs are used for disease detection. Overall, there are 414 different drugs in this data that are used as decision attributes. Due to the limited number of samples and large number of attributes, medications are grouped based on pharmaceutical classifications written in pharmacology books. The designed drug group is approved by a number of physicians and pharmacists. Finally, pharmaceutical drugs are categorized in 60 groups. Then prescriptions are tagged by physicians and pharmacists. The results show that prescriptions are prescribed for 27 different diseases but we decide to focus on ten important diseases and to put the samples of remaining 17 diseases in a class apart. The data is presented in the form of a 1412 * 60 table in which rows represent the prescriptions and columns show drug groups. The data presented in the table is used for the detection of related diseases which are marked ​​by a physician as numbers 1 to 11. In this table, number one is placed in the cells associated with drug groups of each prescription. If a drug fits to two drug groups, number one is placed in the cells of both groups. Also, if two drugs of a prescription belong to a group, in the related cell we place number two.

**Table 1:** The performance of classifiers in detecting diseases

**Other diseases**	**Hypothyroidism**	**Hyperthyroidism**	**Migraine**	**Anemia**	**H. pylori infection**	**Epilepsy and Seizure**	**Hypertension**	**Diabetes**	**Osteoporosis and calcium deficiency**	**Asthma**	Diseases	
94	100	100	93.7	95.4	98.3	91.1	92	99	93.9	81	Decision Tree	Sensitivity
96.2	100	100	94.9	93.8	98.3	93.1	95.5	99	94.9	85.7	Support Vector Machine	
95.8	100	100	94.9	95.4	96.6	93.1	95.5	99	94.9	85.7	Neural Network	
88.9	98.4	95.2	86.1	89.2	96.6	93.1	89.3	92.8	91.9	81.7	Naïve Bayes	
95.8	100	100	97.5	95.4	96.6	93.1	94.6	99	94.9	81	Logistic Regression	
92.2	96.9	95.2	78.5	89.2	98.3	92.1	85.7	87.6	88.9	77	K-Nearest Neighbor	
96.3	99.9	99.9	99.8	99.2	99.9	98.9	99.4	100	99.98	98.6	Decision Tree	Specificity
96.8	100	100	99.99	99.2	100	99.5	99.5	100	99.8	99.2	Support Vector Machine	
96.6	100	99.9	99.9	99.3	99.9	99.6	99.6	100	99.9	99.1	Neural Network	
95.1	100	99.9	98.9	99.3	98.7	98.6	99.1	99.8	99.5	98.4	Naïve Bayes	
95.9	100	99.9	99.4	99.3	99.9	99.7	99.8	100	99.7	99.3	Logistic Regression	
92.1	99.9	99.9	99.5	99.1	99.8	99.2	98.9	99.8	99.5	98.1	K-Nearest Neighbor	
94.1	98.5	98.4	97.4	84.9	98.3	86.8	92.8	100	96.9	85	Decision Tree	
95	100	100	98.7	84.7	100	94	93.9	100	96.9	91.5	Support Vector Machine	Precision
94.8	100	98.4	98.7	86.1	96.6	94.9	95.5	100	98.9	90.8	Neural Network	
92.1	100	98.3	81.9	86.6	77	83.9	89.3	97.8	93.8	83.7	Naïve Bayes	
93.8	100	100	90.6	87.3	98.3	95.9	98.1	100	95.9	91.9	Logistic Regression	
88.1	98.4	98.3	91.2	82.9	95.1	84.9	87.3	97.7	92.6	80.2	K-Nearest Neighbor	

*Classification techniques*

*Decision Tree*

Decision Tree is a simple and fast way for classification. Decision Tree consists of nodes in which attributes are tested. At the end of each branch, the final nodes represent different classes (16). Basic algorithm of Decision Tree is ID3. ID3 algorithm begins with this question: which attribute must be selected for development? A statistical test is used to determine how much each attribute alone is able to classify samples. This statistical test is called Information Gain. For obtaining Information Gain, entropy must be calculated. Entropy specifies the homogeneity or lack of homogeneity of the sample set. With entropy, as a tool for measuring the degree of homogeneity of a set of training examples, we can obtain Information Gain. ID3 continues until all samples belonging to a class or the best Information Gain would not exceed zero. But sometimes noisy data and the low number of samples may lead to over fitting. The C4.5 algorithm is the new version of ID3 algorithm which is a pruning based method to avoid over fitting. This method not only prevents over fitting but also is able to manage missing values (17).

*Support Vector Machine (SVM)*

SVM is basically a binary classifier that separates two classes using a linear boundary. This method is less prone to over-fitting and is suitable for high-dimension data (16). The SVM uses an optimization algorithm to determine the samples that form the boundaries of classes. These samples are called Support Vectors. A number of training samples that have the shortest distance from the decision boundary can be considered as a subset to define decision boundaries and Support Vectors. When classes overlap, separating them via linear decision boundaries will result in some errors. To solve this problem, SVM uses a nonlinear transformation to move data from the original space to a new space with more dimensions. So, in the new space, there are less confrontation between classes (18). 

As previously mentioned, Support Vector Machine is a binary classifier. So when there are more than two classes, it cannot be used directly. In general, for using binary classifiers in multiclass cases several binary classifiers must be designed. The final classifier is achieved by merging single binary classifiers (19).

*Logistic Regression*

Logistic Regression is a linear function for binary classification. This model is similar to linear regression, however, instead of using parameter estimation method, it uses the maximum likelihood estimation method to minimize errors. In addition, in linear regression the answer is achieved by linear combination of independent variables. Nevertheless, Logistic Regression uses linear combination of logit function (1) to estimate the probability of classification in each category. (1) It has always a value between 0 and 1. In this equation $\beta_{i}$ indicates the parameters of the problem that are changed to fit the data and make the best predictions (20).

$\mathrm{Logit}\left(P\right)=\ln\frac{P}{1-p}=\beta_{0}+\sum_{i}\beta_{i}X_{i}$

(1)

As mentioned above, Logistic Regression is used for binary classification. Multinomial Logistic Regression is the generalized version of Logistic Regression that is used to predict the probability of more than two modes. To calculate Polynomial Logistic Regression, it is assumed that there are separate binary regressions as many as the likely outcomes (21).

*Neural Networks*

 The main idea of Neural Network is derived from the performance of the human brain. Each Neural Network consists of a number of neurons that are connected to each other. These neurons and communications between them determine the behavior of the network. Selecting the type of network depends on the nature of the problem. The most common type of Neural Network is Gradient-based Back Propagation network. This network consists of three or more layers of neurons: an input layer, an output layer, and hidden layers. In most cases only a hidden layer is used to reduce the time of computation, especially when the results are satisfying. In these networks, all the neurons of a layer are connected to the neurons of the next layer by a line of communication. A weight ratio is given to any line of communication between neurons. These weights changes by repeating learning process based on inputs and outputs of each step. In each training time, weights change to minimize the response error. Training continues until the sum of error squares reach the minimum (22).

Neural Network is a suitable method for noisy and incomplete data set and it has the capability of managing missing values. This method can generalize obtained rules to similar cases even when there is a little information about features and classes (16).

*Naïve Bayes*

Bayesian networks are graphical models that show the relationship between a set of attributes. This method calculates the probability of the membership of a sample in a class using Bayes theory. One of the benefits of this method is its computing speed. Furthermore, there is no special parameter to optimize or repeat simulations. In addition, its concepts are easily understandable. This method is suitable for large datasets (16).

Naïve Bayes classifier is a simple Bayesian network which assumes the attributes involved in decision making are independent. That is why it is called Naïve. In other words, the Naïve Bayes theory assumes that the impact of the value of an attribute on a class is independent of the value of the other attributes. This assumption is called the conditional independence and has been set to simplify the calculations (23).

Theoretically the error rate of Bayesian classifier is less than the other classification methods. But in practice, because of the conditional independence assumption, it is not always true. In general, Bayesian methods are less accurate than the other methods (16).

*Nearest Neighbor: *The Nearest Neighbor algorithm is one of the simplest methods of data. This method assumes that objects that are close to each other can be in the same category. K-Nearest Neighbor (KNN) is one of the Nearest Neighbor methods that use *K* adjacent neighbors to determine the new object class. Firstly, in this algorithm *K* is determined. Then, the distance of inputting sample from all training samples is calculated. After this, training samples are sorted based on their distance and *K* -Nearest Neighbors are selected. Finally, output of the new sample is determined based on majority vote of *K* nearest samples (24). 

Nearest Neighbor algorithm is easy to understand and implement and has a good speed. Moreover, it is suitable for a data with a high class number. But, with increasing the number of Nearest Neighbors involved in classification, the computational cost of this method increases. This method gives the same weight to all attributes. This may lead to confusion and reduce the accuracy when unrelated attributes exist in the data. To avoid this problem weighted attributes can be used or the noisy samples can be eliminated. In this method, the correct classification of a sample depends on the structure of the local data. On the other hand this method needs large memories. Also due to the lack of a certain method to determine *K*, a validation method is used and this issue leads to an increased computational cost (16).

*Naïve method*

Naïve methods are usually used to evaluate the results of sophisticated methods. We develop a Naïve method that operated based on shared drug groups between each prescription and diseases. To implement the desired Naïve method, first, a table with a dimension of 11×60 is created in which rows show the diseases and columns represent the drug groups. In each row we wrote number 1 in the cells of drug groups that are related to a specific disease, and in the remaining cells we place 0. Then, all prescriptions are read in turn and the numbers of shared drug groups between prescriptions and each disease are obtained based on the designed table. Since there is different numbers of effective drug groups in identifying diseases, comparing the number of shared drug groups did not help to diagnose the correct disease. Therefore, we decide to normalize numbers via Eq. (2). In this equation $a_{\mathrm{ij}}$ represents the number of shared drug groups between prescription *i* and disease *j*, $b_{i}$ is the number of drug groups of prescription *i* and $c_{j}$ is the number of drug groups of disease *j*. 

$m=\frac{a_{\mathrm{ij}}}{\sqrt[{2}]{b_{i}.c_{j}}}$

(2)

After obtaining the normalized numbers, the prescription is tagged with the disease with the largest average.

*Combining Techniques*

In recent years, researchers have tried to figure out what is the best method of combining a group of classifiers to obtain better estimations. In fact, the most important result obtained from the integrity of the data mining algorithms is to increase the precision. Another important advantage of combining data mining algorithms is that the results achieved from combining a few simple algorithms is usually better than a single sophisticated data mining algorithm.

*Voting:* Voting is one of the easiest and most common ways to combine the output of various data mining algorithms. According to this method, to classify a new instance:

- first, the class of instances is determined separately using each classifier
- Then, the final class is determined by majority vote of classifiers.

**Figure 1:**
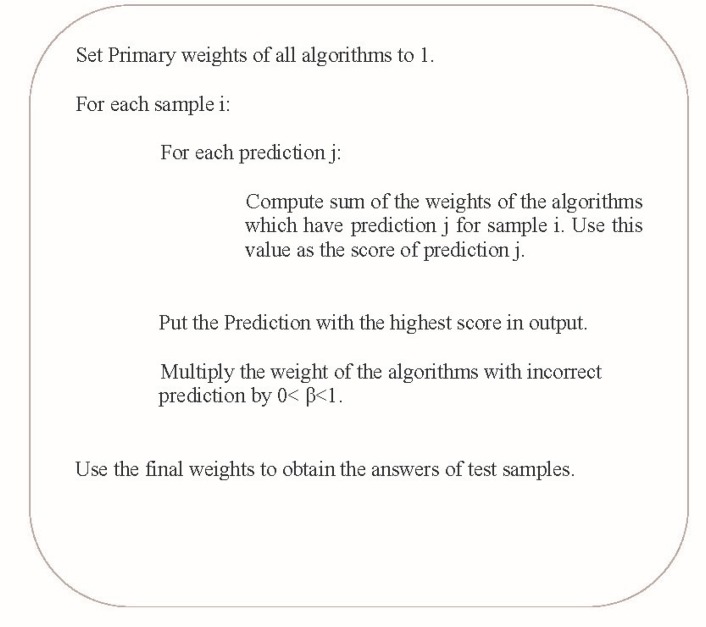
Weighted Voting algorithm

In weighted voting, a weight is given to each classifiers according to its accuracy (25). Figure 1 shows how a weighted voting algorithm works (26).

*Stacking:* Stacking is another combining method that uses an additional layer to combine the outputs of classifiers (27). Suppose *D* is the data and $L^{1}\ldots N^{N}$ is a set of *N* learning algorithms. During the *J-*Fold cross validation process, *D* is randomly divided into *J* different part $D^{1}\ldots D^{j}$with the same size. At every stage *J*, *N* data mining algorithm are trained with $D/D^{j}$ sample and training results are tested. 

The prediction of each algorithm along with actual class of each sample (${\mathrm{MD}}^{J}$) form the next layer of stacking algorithm. At the end of cross validation process forms the complete data of the next layer. This data is given to the data mining algorithm $L^{M}$ for determining the final classes of the data. $L^{M}$can be one of the previous layer algorithms or a different algorithm (25).

## Results and Discussion

We assess the performance of different data mining algorithms in classification of the samples in 11 classes. Then, the results are analyzed and evaluated. In all of the algorithms, 10*-*Fold cross validation method is used for training. The evaluation criteria used in this paper include accuracy, precision, sensitivity, and specificity.

For the implementation of the algorithms, a Decision Tree with minimum leaf size of 1 and Confidence Factor of 0.55 are used. These two parameters specify how to prune the tree. Support Vector Machine used a polynomial function. *C*-factor that determines the amount of margin is set to 1.6. Also Pairwise Coupling is used to implement multi-class Support Vector Machine. A Back Propagation Neural Network with 63 neurons in hidden layer, 60 neurons in input layer and 11 neurons in output layer is used. Learning process is carried out in 500 steps. In this algorithm Learning Rate is set to 0.3 and momentum parameter is set to 0.2. Considering the correlation between the attributes, instead of the simple Logistic Regression, Ridge Regression is used for modeling this correlation. When the Ridge factor is 10, we reach the maximum accuracy. The best accuracy for Nearest Neighbor method is achieved only when one neighbor is used to estimate the output. In this algorithm, Manhattan distance is used as distance metric.

**Figure 2:**
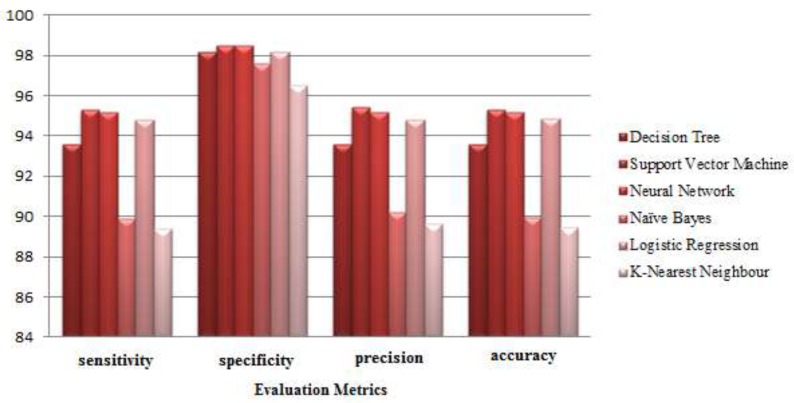
Comparing the classifier performances based on the average evaluation criteria

Figure 2 shows the results of the implementation of data mining methods in the form of a column chart. According to this chart, the total performance of Support Vector Machine is better than the other methods. The results of the implementation showed, when the number of decision attributes is high, the performance of this method is better than the other methods.

Table 2 shows the performance of classifiers. Based on this table, in diagnosis of any disease one of the methods is better than the rest. For example, the Decision Tree method performs well in the detection of Diabetes (No. 3), Support Vector Machines is good in the detection of Helicobacter Infection, Diabetes, Hypothyroidism, Hyperthyroidism, Neural Network in the detection of Osteoporosis and Calcium Deficiency, Diabetes and Hypothyroidism, and finally Logistic Regression shows a good performance in detecting Asthma, Diabetes, Hypertension, Epilepsy and Seizures, Migraines and Hypothyroidism

**Figure 3:**
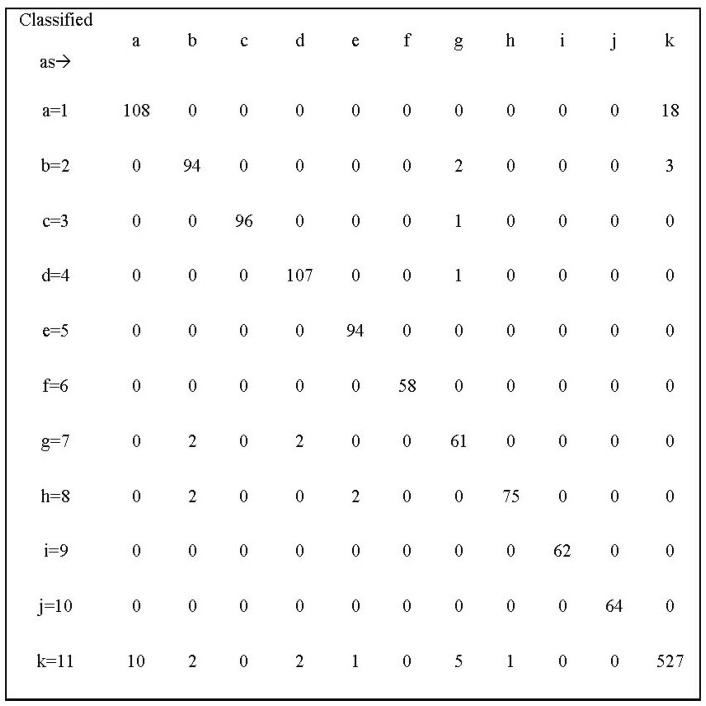
Confusion Matrix of Support Vector Machine

Figure 3 shows the Confusion Matrix of Support Vector Machine. The diagonal of this matrix is the number of samples classified properly for any disease. The frequency of any disease is obtained from the sum of row numbers corresponding to that the disease. As shown, most classification errors occur in class one (Asthma). According to this matrix 14 percent of samples of Asthma are classified as class 11.

If we want to look at this issue from the perspective of physicians, we can say that the incorrect classification of Asthma samples is due to similarities between this disease and Respiratory Infections. For example Anti-Inflammatory Steroid is a common drug group used for both Asthma and Respiratory diseases. Another reason for the incorrect diagnosis of these two diseases is that in recent years there has been an increase in the prescription of anti-Asthma sprays for Respiratory Infection patients. This makes it harder to differentiate these two diseases from each other. All instances of Hypothyroidism and Hyperthyroidism are classified correctly because a few specific medications are prescribed for these two diseases. For example, in 90% of cases Levothyroxine is administered for hypothyroidism and Methymazole is administered for Hyperthyroidism. The errors in the classification of other diseases can be also attributed to the presence of noisy prescriptions. In most prescriptions, in addition to medications of a specific disease, there are one or more non-related medications; these non-related medications might lead to classification error.

**Figure 4:**
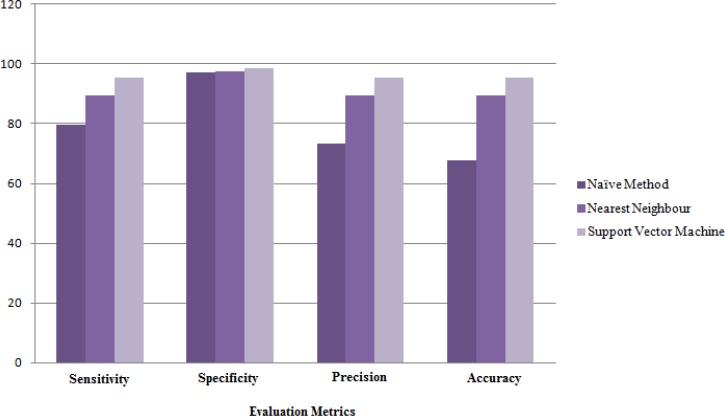
Comparing the Naïve method with data mining algorithms based on average of evaluation metrics

Figure 4 shows the results of Naïve method and also the best and worst results of data mining algorithms. Based on this chart the accuracy of Nearest Neighbor method (with the worst results among the other data mining algorithms) is 16% better than the results of Naïve method. When there are a lot of common attributes between classes, Naïve methods cannot identify the correct class while data mining methods can properly classify the samples by discovering each class pattern.

Since, prescribing methods are different based on the disease stage and patient’s condition, only data mining methods are able to discover these differences. Also in cases where there are a lot of similarities between prescribing patterns of two different disease, data mining methods can properly discriminate the diseases from each other.

Figure 4 shows that there are always better ways to improve results and reduce the rate of error. Accordingly, we decide to improve the results of applied methods by combining the techniques. The results obtained from different data mining methods in the second layer of Stacking algorithm show that among the six algorithms, best results are achieved by the Nearest Neighbor algorithm with three adjacent neighbors. 

**Figure 5:**
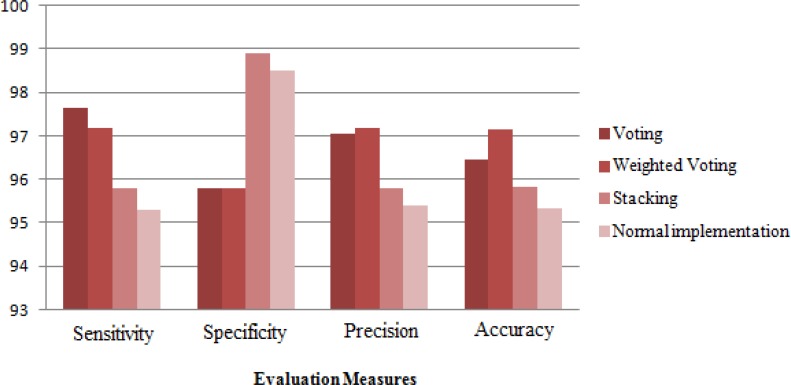
Comparing the performances of combining algorithms

In the weighted voting algorithm to select the updating rate of weights (*β*), all the numbers in the range (0.1:1) with a distance of 0.01 are tested and finally *β* is set to 0.94. The weights of Neural Network, Supporting Vector Machine, Logistic Regression, Decision Trees, Naïve Bayes and Nearest Neighbor methods are equal to 0.395, 0.4759, 0.0085, 0.2129, 0.0132 0.1563, respectively. The accuracy of Support Vector Machine is higher than the other methods; hence the largest weight is assigned to the method by Weighted Voting algorithm. 

In Voting algorithm *β is *set to 1. In other words, in this algorithm the same weight is assigned to all entries.

Figure 5 shows the results obtained by combining algorithms. According to this chart, voting methods shows better performance than Stacking method. Weighted Voting algorithm is less sensitive than Voting algorithm but with an accuracy of 97.16% and precision of 97.19% it could achieve better results.

## Conclusions

In this study a set of data collected from 1412 prescriptions with 414 kinds of drugs is used. In order to reduce the number of decision attributes, medications are classified into 60 drug groups. In this study we decided to focus on ten important diseases. So, the rest of the disease samples are put in a separate class. Classification techniques which are used in this study include Decision Tree, Support Vector Machine, Naive Bayes, Neural Network, Logistic Regression, and Nearest Neighbor, among which Support Vector Machine with an accuracy of 95.32% shows better performance than the other methods. For evaluating the results of the applied classification methods, a Naïve method is implemented. Based on the results, accuracy of this method is 16% worse than Nearest Neighbor methods which have the lowest level of accuracy among the other classification algorithms. In the next stage, combining methods are used to improve the results of the data mining algorithms. These methods include Voting, Weighted Voting, and Stacking algorithms. In all these methods, estimations are obtained from six data mining algorithms and are combined to form the final estimates. Among these combining methods, Weighted Voting algorithms with an accuracy of 97.16% have better performance than the others. 

This study shows that data mining algorithms are suitable algorithms for our classification problem. It is clear that the presence of attributes such as age, gender and specialty of physicians can help to further improve the results and differentiate some diseases easier.

## References

[1] Horton R (2012). GBD 2010: understanding disease, injury, and risk. The Lancet.

[2] Delavari A, Malekzadeh R, Jamshidi HR, Larijani B (2014). NASBOD 2013: Design, definitions, and metrics. Arch. Iran. med.

[3] Molinari NA, Ortega-Sanchez IR, Messonnier ML, Thompson WW, Wortley PM, Weintraub E, Bridges CB (2007). The annual impact of seasonal influenza in the US: measuring disease burden and costs. Vaccine.

[4] Eloand S, Karlberg I (2009). Validity and utilization of epidemiological data: a study of ischaemic heart disease and coronary risk factors in a local population. Public Health.

[5] Orueta JF, Nuño-Solinis R, Mateos M, Vergara I, Grandes G, Esnaola S (2012). Monitoring the prevalence of chronic conditions: which data should we use?. BMC Health Serv. Res.

[6] Desai R, Haberling D, Holman RC, Singleton RJ, Cheek JE, Groom AV, Steiner CA, Parashar UD, Esposito DH (2012). Impact of rotavirus vaccine on diarrhea-associated disease burden among American Indian and Alaska native children. Pediatrics.

[7] Ting S, Wang WM, Kwok SK, Tsang AH, Lee W (2010). RACER: Rule-Associated CasE-based Reasoning for supporting General Practitioners in prescription making. Expert Syst. Appl.

[8] Hannan SA, Bhagile V, Manza R, Ramteke R (2010). Diagnosis and medical prescription of heart disease using support vector machine and feedforward backpropagation technique. Int. J. Computer Sci. Eng.

[9] Toussi M, Lamy JB, Le Toumelin P, Venot A (2009). Using data mining techniques to explore physicians' therapeutic decisions when clinical guidelines do not provide recommendations: methods and example for type 2 diabetes. BMC Med. Inform. Decis. Mak.

[10] Chazard E, Preda C, Merlin B, Ficheur G, Beuscart R (2009). Data-mining-based detection of adverse drug events. MIE.

[11] Hammann F, Gutmann H, Vogt N, Helma C, Drewe J (2010). Prediction of adverse drug reactions using decision tree modeling. Clin. Pharmacol. Ther.

[12] Huang LC, Wu X, Chen JY (2011). Predicting adverse side effects of drugs. BMC genomics.

[13] Visweswaran S, Hanbury P, Saul M, Cooper GF (2003). Detecting adverse drug events in discharge summaries using variations on the simple Bayes model, AMIA Annual Symposium Proceedings, American Medical Informatics Association.

[14] Bate A, Lindquist M, Edwards I, Olsson S, Orre R, Lansner A, De Freitas RM (1998). A Bayesian neural network method for adverse drug reaction signal generation. Eur. J. Clin. Pharmacol.

[15] Botsis T, Nguyen MD, Woo EJ, Markatou M, Ball R (2011). Text mining for the Vaccine Adverse Event Reporting System: medical text classification using informative feature selection. J. Am. Med. Inform. Assoc.

[16] Bhavsar H, Ganatra A (2012). A comparative study of training algorithm for supervised machine learning. Int.J. Soft  Comput. Eng. (IJSCE).

[17] Maimon OZ, Rokach L (2005). Data mining and knowledge discovery handbook.

[18] Gunn SR (1998). Support vector machines for classification and regression. ISIS technical report.

[19] Duan KB, Keerthi SS (2005). Which is the best multiclass SVM method? An empirical study. Multiple Classifier Systems.

[20] Le Cessie S, Van Houwelingen J (1992). Ridge estimators in logistic regression. Applied statistics.

[21] Starkweather J, Moske AK (2011). Multinomial logistic regression. Consulted page at September 10th.

[22] Reby D, Lek S, Dimopoulos I, Joachim J, Lauga J, Aulagnier S (1997). Artificial neural networks as a classification method in the behavioural sciences. Behav. Process.

[23] Rish I (2001). An empirical study of the naive Bayes classifier, IJCAI 2001 workshop on empirical methods in artificial intelligence.

[24] Cunningham P, Delany SJ (2007). k-Nearest neighbour classifiers. Mult. Classif. Syst.

[25] Sigletos G, Paliouras G, Spyropoulos CD, Hatzopoulos M (2005). Combining information extraction systems using voting and stacked generalization. J. Mach. Learn. Res.

[26] Littlestone N, Warmuth MK (1994). The weighted majority algorithm. Inform. Comput.

[27] Wolpert DH (1992). Stacked generalization. Neural Net.

